# Testosterone, territorial response, and song in seasonally breeding tropical and temperate stonechats

**DOI:** 10.1186/s12862-017-0944-9

**Published:** 2017-04-17

**Authors:** Beate Apfelbeck, Kim G. Mortega, Heiner Flinks, Juan Carlos Illera, Barbara Helm

**Affiliations:** 1 Institute of Biodiversity, Animal Health and Comparative Medicine, University of Glasgow, Glasgow, Scotland G12 8QQ UK; 2 Terrestrial Ecology Research Group, Department of Ecology and Ecosystemmanagement, Technische Universität München, School of Life Sciences Weihenstephan, D-85354 Freising, Germany; 3 Department of Migration and Immunoecology, Max-Planck-Institut für Ornithologie, D-78315 Radolfzell, Germany; 4 Am Kuhm 19, D-46325 Borken, Germany; 5 Research Unit of Biodiversity (UO-CSIC-PA), Oviedo University, Campus of Mieres, 33600 Mieres, Spain

**Keywords:** Steroid hormones, Testosterone, Territorial behavior, Migratory behavior, Song, Tropical, Temperate, Songbirds, Breeding season length

## Abstract

**Background:**

Testosterone facilitates physiological, morphological, and behavioral changes required for breeding in male vertebrates. However, testosterone concentrations and the link between its seasonal changes and those in reproductive behaviors vary greatly among species. To better understand the impact of tropical and temperate environments and life history factors on this variation, we have compared testosterone, territorial behavior and song performance across sequential stages of the breeding season in males of 16 closely related taxa of East African tropical and West European temperate stonechats (*Saxicola* spp), which all breed during a short breeding season, but differ in migratory behavior, seasonal territory-acquisition and pace of life.

**Results:**

We found that generally, the profiles of testosterone and territorial behavior were similar across latitudes. African stonechats with a slow pace of life had equally high peak testosterone concentrations and responded as aggressively to an intruder as European stonechats with a fast pace of life. However, song performance at the beginning of the breeding season was lower in African than in European stonechats. The differences in song performance were not associated with variation in testosterone levels between tropical and temperate stonechats.

**Conclusions:**

The results suggest a very similar role for testosterone as a mediator of high intensity territorial aggression during the fertile period of females in tropical and temperate stonechats, which all are highly seasonal, locally synchronous breeders. A potential explanation may be high risk of extra-pair copulations which has been associated with synchronous breeding. Interestingly, an association was not consistent for song performance. Our data suggest that song performance can be disassociated from peak testosterone levels depending on its role in breeding behavior. Despite similar testosterone levels, European males, which early in the breeding season acquire territories and mates, showed greater song performance than African stonechats, which maintain year-round territories and pair-bonds. Taken together, our study comparing related taxa of old world songbirds suggests that short breeding seasons may be a major selective force for high peak testosterone levels during breeding regardless of latitude and pace of life, but that particular behaviors, in our case song, can be uncoupled from peak testosterone levels.

**Electronic supplementary material:**

The online version of this article (doi:10.1186/s12862-017-0944-9) contains supplementary material, which is available to authorized users.

## Background

The sex steroid testosterone coordinates many reproductive traits in male vertebrates (reviewed by [1]). Testosterone has pleiotropic effects and enhances male fecundity by activating spermatogenesis, secondary sex characteristics and sexual and aggressive behaviors associated with breeding (reviewed by [1, 2]). These effects are supported by experimental studies and by coinciding seasonal profiles of testosterone with reproductive behaviors. For example, implants of testosterone can increase territorial aggression (e.g. [3, 4]) and song rate (e.g.[5, 6]), while implants of androgen receptor blockers can decrease territorial aggression (e.g. [7–10]) and the occurrence of dawn song [11]. Coinciding seasonal profiles of testosterone and behavior have been observed in detail in many temperate seasonally breeding birds. For example in temperate socially monogamous birds, males commonly engage strongest into territorial disputes (e.g. [12–15]), sing most (e.g. [16–21]) and have the highest testosterone levels at the beginning of the breeding season when territories are established and females are fertile (e.g. [21–25]).

However, quantitative differences in peak testosterone levels and differences in the degree to which testosterone mediates reproductive behaviors have been observed [26]. For example, many tropical species with extended breeding seasons tend to have lower testosterone levels than high latitude species with short breeding seasons [27–30]. Two main explanations have been suggested for what had seemed to be a general pattern of lower testosterone concentrations in tropical birds. Firstly, assuming generally longer breeding seasons in the tropics, lower testosterone levels may help to avoid extended costs associated with testosterone, such as potential impairment of the immune system, survival and male parental care [31, 32]. Alternatively or in addition, tropical species typically fall on the “slow” end of the pace of life syndrome [28, 33] by being longer-lived, showing reduced metabolic measures and lower fecundity, often paired with extended parental care. Thus, they may especially benefit from low testosterone concentrations throughout the breeding season, which would aid survival while imposing little cost given low fecundity.

More recently, however, several studies have shown that males of tropical species that breed in highly seasonal environments have indeed similar peak testosterone concentrations as temperate seasonal breeders ([34–38]; reviewed by [29, 39]). It has, therefore, been suggested that seasonal peak testosterone levels are determined by the length of the breeding season and associated factors independently of breeding latitude [29]. Among the factors that are associated with different degrees in seasonality and that could influence peak testosterone levels is type of territoriality, depending on seasonal migration [39]. Permanent residents, which are more common in the tropics, often defend year-round territories and form long-term monogamous pair bonds regardless of the timing of breeding [40], whereas migratory species typically acquire territories and breeding mates after returning to the breeding grounds [41]. A second factor that has been associated with different degrees in seasonality and that could influence peak testosterone levels is risk of extra-pair copulations, which is thought to increase when breeding seasons are short and pairs are highly synchronous [40, 42–44]. Thus, a perceived pattern of low peak testosterone in the tropics may have resulted from heavy emphasis on species breeding in the lowland forests of the Neotropics with low seasonality and extended breeding seasons. In these habitats, low risk of extra-pair copulations and typically continuous territory ownership could have specifically selected for low testosterone levels [40, 42]. Thus, based on hormone profiles, tropical seasonal breeders could be expected to show similar territorial behavior and song performance as temperate seasonal breeders.

In contrast, based on life-history patterns, it could still be expected that tropical and temperate seasonally breeding species differ in their testosterone profiles and in the behaviours that are commonly under testosterone control. Even in seasonal tropical biomes some environmental conditions are relatively stable year-round (e.g. only minor changes in temperature and day length [45, 46]). Such general tropical-temperate differences seem to have promoted a slow pace of life in the tropics with reduced fecundity and increased survival [33, 47], which for birds has been robustly established. For example, tropical birds, independent of biome, lay smaller clutches [48] and have lower basal metabolic rates [49], but live longer [50, 51] than temperate birds. Based on their low fecundity and greater longevity, tropical species generally are expected to invest less into mating than temperate species [28, 52]. Thus, while apparently overall differences in peak testosterone concentrations appear to depend on the length of the breeding season, pace-of-living could be associated with differences in the fine seasonal profile of testosterone levels, or in the association of testosterone with behaviours linked to specific aspects of breeding, such as territorial aggression and song. For example, it has been proposed that in birds with year-round territories, but short, synchronous breeding seasons, the major role of testosterone is to promote territorial aggression and mate-guarding during the fertile period of females to prevent extra-pair copulations [39].

In birds, song is a major component of territory establishment and mate attraction [53–55] and different song characteristics matter in these different contexts [56, 57]. For example, high element rates have been described in courtship songs of several species and seem to be sexually attractive to females when tested under laboratory conditions (e.g. [58, 59]). Song rate has been shown to increase after the experimental removal of the mate [54] and in many species females prefer males that sing at high rates (e.g. [60]). Species that are supposed to be under greater pressure to establish a territory and attract a mate at the beginning of the breeding season are expected to sing more and more complex song than species breeding under more relaxed conditions [61–63]. Therefore, non-withstanding the general similarity in testosterone profiles, song characteristics are expected to differ between high-paced temperate and slow-paced tropical species which have low fecundity and no need for annual acquisition of territories and mates. Indeed, comparative studies across latitudinal gradients find that migratory species, species with high annual fecundity and species breeding at higher latitudes sing longer songs, with more syllables and at a higher rate than non-migratory resident species, species with low fecundity and species breeding closer to the equator [64–69]. Despite this body of literature, the implications of similar peak testosterone concentrations in tropical and temperate seasonal breeders for the variation of behaviors related to mating are still unclear. In addition to these research gaps, there is need for a broader coverage of global biomes and for insights from closely related species. Most of our present knowledge on testosterone, song and territorial behavior in tropical birds is biased towards species breeding in the lowland forests of the Neotropics with low seasonality and extended breeding seasons, which are often compared to different Nearctic species.

In this study, we therefore compare testosterone, song and territorial behavior during different breeding stages within a group of closely related old world songbirds. Stonechats (*Saxicola* spp) have a wide distribution range from tropical to temperate environments, but wherever they occur, they are seasonal breeders [70]. Many other traits differ between stonechats from different environments. For example, they display a full range of migratory habits from long-distance migrant to resident [70], and they are a classical example for differences in pace of life between tropical and temperate birds as highlighted in Ricklefs’ and Wikelski’s influential article in 2002 [33]. The behavioral endocrinology of temperate and tropical stonechats is also well studied. During the breeding season, territorial behavior in temperate stonechats has been shown to depend on testosterone [8, 71] and individual variation in testosterone concentrations during mating, but not during parenting, relates to variation in reproductive success [72]. However, temperate male stonechats do not show short-term increases in testosterone concentrations during simulated territorial intrusions despite an aggressive response towards the intruder [73]. Furthermore, territorial aggression outside the breeding season is independent of testosterone [8]. Thus, in temperate stonechats testosterone may mediate aggressive behavior particularly in a mating context and may promote aggressive mate-guarding during the fertile period of females. In tropical stonechats, Dittami and Gwinner [74] studied seasonal patterns in reproductive hormones, and found weak, annual patterns in testosterone and LH in Kenyan males [74]. They found indication of declines in both hormones during the breeding season, but had low power for fine-scale resolution of breeding seasonality. In a more detailed study, Goymann et al. [36] compared testosterone of tropical Kenyan male stonechats during the breeding season to those of temperate German stonechats [36]. They found that African stonechats showed a pronounced peak of testosterone during the nest-building and egg-laying stage. Compared to European stonechats, the study reported overall lower testosterone in African stonechats, except for the nest-building and egg-laying stages. Associations between the seasonal profile of testosterone and aggressive and vocal behaviors are currently not known for tropical stonechats.

Few studies so far have considered song within the comparative stonechat system. Earlier work in temperate stonechats showed that song plays an important role to attract a mate [12, 75] and song rate peaks at the beginning of the breeding season, just before egg-laying of the first brood [12]. Detailed seasonal song profiles are not available for tropical stonechats, however both tropical and temperate stonechats only sing during the breeding season [74, 76]. An earlier study on temperate stonechats has studied song performance and aggression in response to territorial intrusions using song playback and decoys [77], but did not relate these findings to hormone concentrations.

Building on the strength of the comparative stonechat system, we extend the earlier work to compare testosterone, territorial behavior and song across a wide range of tropical and temperate stonechat populations. The broad latitudinal comparison is intended to produce generalizable conclusions, while the combination with detailed territorial and song behavior is entirely novel. We compare populations of East African tropical and West European temperate male stonechats that differ in fecundity, territoriality and the stability of pair bonds. Specifically, our study aims

i) to test the generality of previously reported similar testosterone peaks using males of several populations, during sequential stages of the breeding cycle.
ii) to test whether seasonal changes in testosterone are associated with *population-wide* changes in territorial aggression and song performance. As male temperate, but not tropical, stonechats, have to establish a territory and attract a female at the beginning of the breeding season, we expect higher territorial aggression and higher song performance (song rate, element rate, peak frequency) in temperate than tropical stonechats at the beginning of the breeding season. We expect that a lack of differences would indicate that testosterone in tropical stonechats has other functions, such as mediating aggression in a mating context. If so, we predict that both testosterone and aggression should peak in both tropical and temperate stonechats during nest-building when females are fertile.

## Methods

### Study system

#### Species background

Stonechats are especially well suited to address the aims specified above, because we have extensive knowledge of the life history and endocrinology of tropical and temperate populations. Tropical stonechats follow a slower pace of life than temperate stonechats. They have lower fecundity, fewer annual clutches and a lower metabolic rate than their higher latitude relatives. Common garden experiments have shown that these differences are genetically fixed [78–80]. Despite these and other differences in pace of life, stonechats are socially monogamous seasonal breeders with relatively short breeding seasons, and defend a breeding territory regardless of the latitude at which they breed [70]. Tropical stonechats are usually resident and stay with the same mate year round [74]. Migratory temperate stonechats establish separate territories in breeding and wintering areas and form seasonal pair bonds with different mates [76]. Resident temperate stonechats may show local movements during winter depending on environmental conditions [70]. In temperate stonechats, territorial disputes between males are most frequently observed at the beginning of the breeding season [12]. At this time, breeding territories are being established in temperate stonechats, whereas in tropical stonechats establishment of breeding territories occurs after the post-fledgling period [81]. As in other songbirds [82], copulation usually takes place 1–4 days before the first egg is laid [70].

#### Field work

We measured testosterone levels in several populations of tropical East African and temperate European stonechats during different stages of the breeding cycle and studied seasonal changes in territorial behavior and song in a subsample of these populations. During their respective breeding seasons, we studied closely related stonechat species in East Africa (*Saxicola torquata* *axillaris*, 10 populations, 173 individuals, latitudes 0° - 4°S, altitudinal range: 1376–2500 m asl, in the years 2012–2013) and in Europe (*Saxicola torquata rubicola,* 6 populations, 201 individuals, latitudes 37° - 51°N, altitudinal range: 15–1350 m asl*,* in the years 2009–2013). Both in tropical and temperate environments stonechats breed in open shrub and grassland habitats, sometimes close to human habitations. Two of the European populations (Germany: Düffel and Heubach) were sampled as part of a different study on the effects of simulated territorial intrusions on testosterone and behavior [73]. The East African populations are resident and breed at different times of the year corresponding to the two major rainy seasons: Southern populations breed from October to December (Tanzania: Arusha National Park, 3°S, Monduli, 3°S, Pare Mountains, 4°S, Usambara Mountains, 4°S), while Northern populations breed from March to July (Kenya: Kinangop, 0°N, Olkalou, 0°N, Mount Kenya, 0°N, Ngong Hills, 1°S, Kakamega, 0°N, Mataara, 0°S). European stonechats breed from March to July and differ in migratory disposition. Three of the sampled European populations were migratory (Western (Düffel and Heubach, 51°N) and Southern (Bavaria, 47°N) Germany), two were partial migrants (Central Spain, 40°N, Ireland, 51°N) and one was a resident population (Southern Spain, 37°N). Breeding stage was determined through careful observation of pairs before capture (e.g. singing activity, nest-building activity by females, feeding activity of nestlings, the presence of fledglings). In some cases, we were able to catch the female and breeding stage could be ascertained by the presence or absence of a brood patch. For located nests, we recorded the number of eggs or nestlings and checked the nest again after a few days. However, especially when females are egg-laying or incubating, it can be very challenging to determine the exact breeding stage (when unable to catch the female), thus for a number of pairs we were not able to ascertain this information. We determined the age of all individuals (yearling or adult (≥ 2years)) based on the wing molt pattern [83] and a picture of the wing was taken for later reference. All birds were bled, measured (body mass, tarsus and wing length), checked for molt, ringed with a uniquely numbered aluminum ring and a combination of three color plastic rings, and released back into their territories.

### Capture methods

Males were caught between 7:00 h and 18:00 h with baited clap net traps and/or clap net traps combined with a mounted decoy and playback. In Europe, we used playbacks from British or Spanish stonechats downloaded from the British library or www.xeno-canto.org, respectively. In East Africa, we used playbacks recorded from the same population. As decoys we used stuffed males in full adult plumage that were protected by an inconspicuous cage. In Europe, we randomly assigned two decoys from Austria and in East Africa eight decoys from each of the two populations, Kinangop (Kenya) and Arusha (Tanzania). In stonechats, stimulation with a decoy and playback does not change circulating testosterone concentrations [72], and hence to analyze testosterone profiles data from passively and actively caught birds were combined.

### Blood sampling and hormone analysis

Immediately upon capture (mean ± SD: 228 ± 191 s), a blood sample (~ 120 μl) was taken after venipuncture from the wing vein and collected into heparinized capillaries. Plasma was immediately separated by centrifugation with a Compur Minicentrifuge (Bayer Diagnostics) or a Spectrafuge Mini Laboratory Centrifuge (Labnet International, Inc.). The amount of plasma was measured with a Hamilton syringe and was stored in 500 μl pure ethanol [84]. After the end of each field season (≤ 4 months) samples were stored at −80 °C. Testosterone concentration was determined by direct radioimmunoassay (RIA, following [36] and [85]). Mean efficiency of the extraction with dichloromethane varied between 81% and 89%. Samples were measured in duplicates in five assays. Three of the assays contained both samples from African and European populations. Samples that had been obtained in Germany in 2009 and 2010 had been analyzed separately in the same laboratory using the same assays [73]. The intra-extraction coefficients of variation ranged between 2.8% and 11.8% (mean 5.6%). The inter-assay variation was 11.9%. The lower limit of detection of the assay was determined as the first value outside the 95% confidence intervals for the zero standard (Bmax) and was on average 0.35 ± 0.05 pg/tube. As the testosterone antibody (Esoterix Endocrinology) shows significant cross-reactions with 5a–dihydrotestosterone (44%) our measurements may include a minor fraction of this additional androgen.

### Behavioral response to simulated territorial intrusions

For two migratory neighboring populations in Europe (Germany: Düffel and Heubach, *n* = 55) and four populations in Africa (Tanzania: Monduli, Kenya: Kinangop, Olkalou, Mount Kenya, *n* = 46) we performed simulated territorial intrusion (STI) experiments. We recorded the behavior of male stonechats towards an intruder during breeding, using latency to attack as the main metric, because previous experiments in stonechats have shown that other behavioral measures (e.g. response and approach latency) strongly correlate with the latency to attack the decoy [73]. To elicit a territorial response we placed a stuffed decoy into the center of a territory (determined by singing posts of the male or nest location) and played back song. For all tropical tests, we used exclusively decoys of African stonechats, and for all temperate tests, we used exclusively decoys of European stonechats, which all originated from a large pool of a captive breeding program at the German Max Planck Institute for Ornithology [86]. African stonechats were taken as nestlings from several study sites in the Kenyan Rift valley and Arusha National Park (Tanzania), while European stonechats were collected as nestlings from a population in Lower Austria. All birds were raised and bred at the Max Planck Institute. Specimens from the original and subsequent generations were kept in a freezer immediately after natural death. In 2011, Mortega chose those birds for preparation of African decoys which were in full adult plumage and physically best maintained while trying to cover the natural variation in their morphology with specific focus on breast patch patterns within each population. All decoy birds were made by one technical assistant of the Max Planck Institute for Ornithology. Decoys of European stonechats had been chosen and prepared at an earlier date using similar criteria, but had never been used in experiments before and were, thus, in very good condition. All decoys, African and European, were presented in a standardized posture, resembling a perching male stonechat. During experiments we prevented the damage of decoys by protecting them with an inconspicuous cage made of a wire frame and a mist net. The Kenyan taxidermic preparations were employed in STI experiments in the three Kenyan populations that breed from March to July (distance between populations ranged between 50 – 95 km) and the Tanzanian decoys were used in the Tanzanian population that breeds from October to December similar to the population where the decoy was from (distance between populations 40 km). For song playback, in Germany, we used eight different playbacks in random order (wav - files, British library). In East Africa, we used four to six different playbacks recorded from the same population and took care to avoid songs from neighboring males within hearing distance. Territory holders were observed until they attacked the decoy (min), but not longer than 60 min. Therefore, males that did not attack the decoy were assigned an attack latency of 60 min.

### Song recording and analysis

We recorded 30–45 min of song from males of the same two European (8:00 h - 12:00 h, *n* = 30) and seven East African populations (5:20 h - 10:00 h, *n* = 32) using a Marantz PMD 661 solid state recorder (Osnabrück, Germany) and Sennheiser ME66/K6 directional microphones (Georgsmarienhütte, Germany). In general, 5–10 min of song were of sufficient quality to be analyzed. We analyzed songs (sampling frequency: 44.1 kHz; resolution: 16 bit) using the software Avisoft Sound Analysis Pro, v5.1.09 (Raimund Specht, Berlin, Germany). We considered five parameters to describe song output and structure in our study species [77]: song rate (average number of songs per minute over a five minute period), element rate (number of elements divided by song duration), as well as peak (frequency of the highest amplitude sound), minimum, and maximum frequencies. For a spectrogram of an exemplary song in European stonechats see [77]. We used the automatic parameter measurements setup to obtain the minimum and maximum frequency values measured at a standard decibel threshold (here −20 dB, total option) below the peak in the power spectra [87]. We compared song between African and European stonechats during the pre-nesting and nest-building stages.

### Statistical analysis

Data were analyzed with the program R (R version 3.2.2, [88]), and using the packages JAGS [89] and runjags [90]. We chose a Bayesian approach to draw inferences from general linear mixed models. We determined whether testosterone concentrations, latency to attack an intruder and measures of song activity and structure differed between stonechats breeding at different latitudes (tropical or temperate) and breeding stages (a factor with up to six levels: pre-nesting, nest-building, egg-laying, incubation, feeding of nestlings, and feeding of fledglings, see below) and the interaction between latitude and breeding stage. In all models, we included population identity as a random intercept. Model parameters were estimated as the mean of their posterior distributions, and the 2.5% and 97.5% credible intervals. We used minimally informative priors for both mean (dnorm (0, 10^^−6^)) and variance (dgamma (0.001, 0.001)) parameters and MCMC simulations were run in two parallel chains for each parameter. Covariate predictor variables (time of day, body mass) were centered to a mean of zero and were removed from the models when they did not explain a detectable amount of variation in the data (i.e. Bayesian credible intervals included zero). Predictor variables that were part of the experimental set-up/hypothesis were always retained in the models. MCMC simulations were checked for convergence of chains using trace plots and psrf values [91]. Effective sample sizes were > 15000 in all cases. Model residuals were graphically checked for violations of model assumptions (normality, heteroscedasticity, autocorrelations). Sample sizes for the different measures differed, as for some individuals we obtained data on testosterone, behavior and song, while for other individuals we were only able to obtain one of these measures.

Testosterone levels were natural log-transformed prior to analysis and are reported on a natural log scale unless stated otherwise. In a first step, we analyzed all testosterone samples (*n* = 357) including also individuals without exact breeding stage information to determine the effect of latitude on testosterone levels. We categorized breeding phase as early, medium or late depending on sampling date and the exact breeding stage of some individuals and a less exact estimate in others. To test whether the relationship between breeding phase and testosterone differs between tropical and temperate latitudes, we included an interaction between latitude and breeding phase in the model. Furthermore, we controlled for capture method (playback yes/no), time of day, body mass, and age. In a second step, we reduced the data set to include only individuals with known breeding stage (*n* = 299). Because European stonechats have a longer breeding season, we only used data from the first brood to match the typical single-broodedness of African stonechats. As time of day at capture, capture method and body mass had no detectable influence on testosterone in the larger data-set (see below) we did not include them in this model.

Attack latency, song rate and frequency parameters were natural log-transformed prior to analysis. Breeding stage was categorized as a factor with four levels (pre-nesting, nest-building, egg-laying and incubation, feeding of nestlings and fledglings). We analyzed whether the latency to attack a simulated territorial intruder differed between males from Africa and Europe, considering breeding stage or the interaction of latitude and breeding stage. Lastly, we analyzed whether song rate, element rate and frequency parameters differed between African and European stonechats during combined pre-nesting and nest-building phases. As the models concerning behavioral data also included individuals that we were not able to catch, no other factors (e.g. age, body mass or capture method) were included in these models.

## Results

### Morphology

As expected from previous studies [92], stonechats from Africa were larger than stonechats from Europe (posterior means and credible intervals; tarsus length: Africa: 22.9 mm [22.8, 23.0], Europe: 22.4 mm [22.1, 22.6], difference: −0.5 mm [−0.7, −0.4]; wing length: Africa: 71.5 mm [71.3, 71.8], Europe: 68.2, [67.5, 68.9], difference: −3.4 mm [−3.8, −2.9]) and heavier (Africa: 16.2 g [16.1, 16.4], Europe: 14.5 g [14.1, 14.8], difference: −1.8 g [−2.0, −1.6]).

### Testosterone levels

**Fig. 1:**
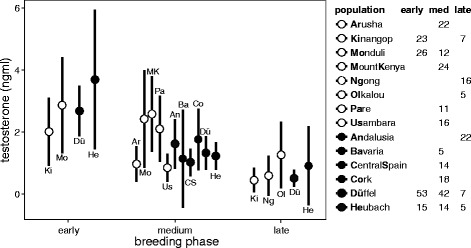
Testosterone concentrations (ng/ml) of African and European male stonechats in relation to breeding phase and population. Points and error bars (means and 95% credible intervals) represent African (white fills) and European (black fills) populations. Letters indicate the location of each population and full location names are displayed together with sample sizes in the legend

When all data were considered, testosterone concentrations were highest early in the breeding season and then declined gradually. This effect of breeding phase on testosterone concentrations was similar at different latitudes, and testosterone did not differ between tropical and temperate environments (Table 1, Fig. 1). Time of day (slope: −0.0002 [−0.0006, 0.0003]), capture method (difference playback no – yes: −0.2 [−0.5, 0.07]) and body mass (slope: 0.01 [−0.15, 0.17]) had no detectable influence on testosterone concentrations. Yearling males had lower testosterone levels than adult males (difference adult - yearling: -0.30 [-0.58, -0.02]).

**Table 1:** Estimates of natural log-transformed testosterone concentrations of male stonechats in relation to latitude and breeding phase

	Estimates and 95% credible intervals	Estimates and 95% credible intervals	Estimates and 95% credible intervals	
	Africa (intercept)	Europe	Difference Africa – Europe	
Breeding phase				
Early (intercept)	7.18 [6.84, 7.51]	7.48 [6.74, 8.24]	0.31 [-0.12, 0.73]	
Medium	6.74 [6.11, 7.37]	7.05 [6.0, 8.1]		
Late	5.83 [5.05, 6.62]	6.13 [4.94, 7.35]		

Columns show Bayesian estimates and credible intervals. When zero is not included within the credible intervals of the differences there is a detectable effect of this parameter on the dependent variable (printed in bold).

**Fig. 2:**
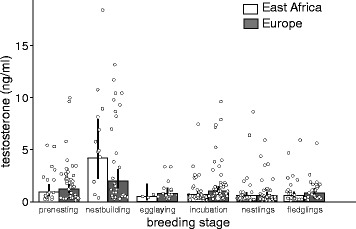
Testosterone concentrations (ng/ml) of African and European male stonechats during detailed breeding stages of the first brood. Especially in African stonechats, testosterone concentrations peaked during nest-building and were lowest when stonechats were feeding nestlings. Bars and error bars represent back-transformed posterior means and their 95% credible intervals. Dots represent data points of individuals from different populations. Populations were included as random intercepts in the models

The model including only individuals for which exact breeding stage was known, revealed that at all latitudes testosterone concentrations were especially low during the nestling stages (Table 2, Fig. 2). They peaked during nest-building, but this peak appeared to be more pronounced in African than European stonechats (Table 2, Fig. 2). Within this data-set, testosterone concentrations did not differ between age classes (difference adult – yearling: −0.22 [−0.51, 0.07]).

**Table 2:** Estimates of natural log-transformed testosterone concentrations of male stonechats in relation to latitude and detailed breeding stage

	Estimates and 95% credible intervals	Estimates and 95% credible intervals	Estimates and 95% credible intervals
Breeding stage	Africa (intercept)	Europe	Difference Africa – Europe
Pre-nesting (intercept)	6.96 [6.46, 7.46]	7.25 [6.15, 8.35]	0.29 [−0.31, 0.89]
Nest-building	8.36 [7.07, 9.64]	7.46 [5.59, 9.68]	***-0.9 [−1.84, 0.04]***
Difference	**1.39 [0.61, 2.18]**	0.21 [−0.56, 1.33]	
Egg-laying	6.33 [4.49, 8.13]	6.54 [3.26, 9.79]	0.21 [−1.23, 1.66]
Difference	−0.66 [−1.97, 0.66]	−0.71 [−2.89, 1.44]	
Incubation	6.58 [5.44, 7.7]	6.83 [4.91, 8.74]	0.25 [−0.53, 1.04]
Difference	−0.38 [−1.02, 0.25]	−0.42 [−1.24, 0.39]	
Nestlings	6.32 [5.16, 7.48]	6.26 [4.27, 8.26]	-0.06 [−0.89, 0.78]
Difference	*−* ***0.64 [−1.3, 0.02]***	**−0.99 [−1.88, −0.09]**	
Fledglings	6.4 [5.23, 7.56]	6.55 [4.53, 8.56]	0.15 [−0.7, 1.00]
Difference	−0.56 [−1.21, 0.1]	−0.7 [−1.62, 0.21]	

Columns show Bayesian estimates and credible intervals. Differences refer to differences from the pre-nesting intercept. When zero is not included within the credible intervals of the differences there is a detectable effect of this parameter on the dependent variable (printed in bold). Italic font indicates credible intervals that cross zero only marginally.

### Behavioral response to simulated territorial intrusions

**Fig. 3:**
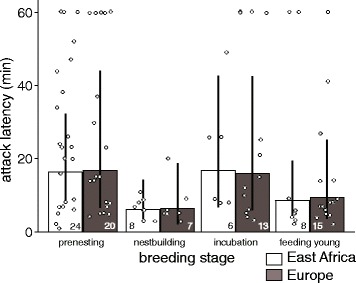
Latency to attack (min) of African and European male stonechats in response to simulated territorial intrusions during different stages of the breeding season. Male stonechats attacked the decoy faster during nest-building and parental stages than during pre-nesting and incubation. Males that did not attack the decoy were assigned a latency of 60 min. Bars and error bars represent back-transformed posterior means and their 95% credible intervals. Dots represent data points of individuals from different populations

Latency to attack a decoy did not depend on latitude (natural log-transformed difference Africa – Europe: 0.04 [−0.35, 0.44], Fig. 3), and changed similarly with breeding stage in tropical and temperate environments (intercept pre-nesting: 2.8 [2.4, 3.1], difference nest-building: −1.0 [−1.5, −0.4], incubation: 0.03 [−0.6, 0.5], feeding of nestlings and fledglings: −0.6 [−1.1, −0.1]). Males attacked the intruder faster during nest-building and parental stages than during pre-nesting (Fig. 3).

### Song

**Fig. 4:**
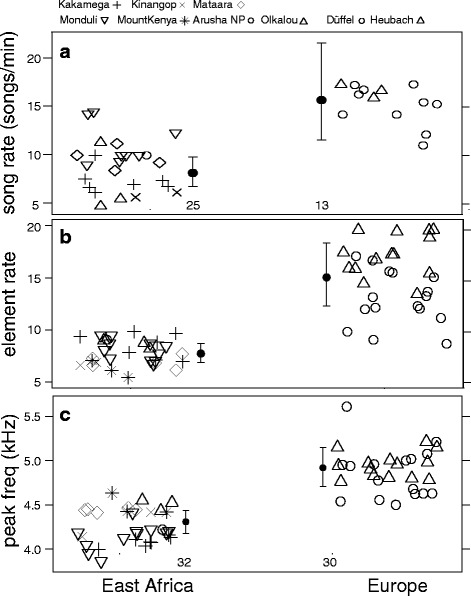
Song traits of African and European stonechats with **a**) song rate, **b**) element rate per song, and **c**) peak frequency. European stonechats sang with a higher song rate, element rate, and peak frequency during the mating period than African stonechats. Filled points and error bars represent back-transformed posterior means and 95% credible intervals. Sample sizes are displayed below error bars and are similar for element rate and peak frequency. Open symbols represent data points of individuals from different populations

Several song traits differed between African and European stonechats during the mating phase (pre-nesting and nest-building). European stonechats sang with a higher song rate (natural log-transformed difference Africa – Europe: 0.64 [0.27, 1.01]), element rate (0.66 [0.43, 0.9]), and peak frequency (0.13 [0.07, 0.19]) than African stonechats (Fig. 4). Minimum and maximum frequencies did not differ between African and European stonechats (natural log-transformed difference Africa – Europe: 0.15 [−0.12, 0.42]; 0.08 [−0.009, 0.17]).

## Discussion

This comparative study on stonechats revealed that despite their slow pace of life, but in accordance with their seasonal breeding behavior, males of several populations of tropical African stonechats expressed similar peak concentrations of testosterone as male temperate European stonechats. These findings are in accordance with results from meta-analyses that suggested that tropical and temperate seasonal breeders express similar peak testosterone concentrations and that the length of the breeding season may be a better predictor of maximum testosterone concentrations than latitude [29, 39]. Our findings also generally confirm earlier data on temperate and tropical stonechats, which demonstrated seasonal peaks during nest-building when females are fertile [36, 74]. In contrast to Goymann et al. [36], our more extensive study found no evidence for overall lower levels of testosterone in tropical compared to temperate stonechats.

In our study, seasonal changes in testosterone were similar in tropical and temperate stonechats and were associated with seasonal changes in territorial aggression: both temperate and tropical stonechats had the highest testosterone levels and were most aggressive during nest-building – the period of highest female fertility. In contrast, song performance at the beginning of the breeding season was lower in African than in European stonechats and differences in song were not associated with variation in testosterone levels between tropical and temperate stonechats. This indicates that breeding season length has a stronger influence on testosterone and the expression of aggressive behaviors than the type of territoriality or pace of life. Short seasonal breeding seasons usually lead to more synchronous breeding with more competition between males and potentially a higher risk of extra-pair copulations (breeding synchrony hypothesis, [93]). Synchrony is generally high in the stonechats we studied. For example, a previous study on African stonechats in Arusha National Park (which was also one of our study populations) found that stonechats bred during a short breeding season from mid-October to January and initiated laying within 2–4 weeks. Replacement clutches were initiated within 10 days after the loss of a nest [94]. The breeding season of European stonechats is a little bit longer, usually from mid-March/April to July [95]. Replacement of clutches occurs also within 10 days [96]. Thus, because stonechats breed during a short breeding season and replacement clutches occur in fast succession there are always many nests active at the same time. Therefore, extra-pair paternity may pose a high risk to males during the fertile period of their mates at all latitudes, and increased territorial aggression during this period may help to prevent extra-pair copulations. For example, in an experimental study in dark-eyed juncos *(Junco hyemalis)*, testosterone treated males suffered fewer losses of paternity than control males [97]. Supporting the fitness relevance of testosterone during mating, earlier studies in European stonechats found that variation in testosterone concentrations during mating, but not during parental stages, was associated with reproductive success in temperate stonechats [72]. Similar to stonechats, testosterone concentrations also peaked during nest-building and mate guarding in temperate barn swallows *(Hirundo rustica,* [20]), blue tits (*Cyanistes caeruleus,* [16]) and in tropical Seychelle warblers *(Acrocephalus sechellensis,* [98]). In stonechats and other species, territorial behavior outside the reproductive phase, does not appear to be mediated by testosterone. Both tropical and temperate stonechats also defend territories in a non-breeding context. However, antiandrogen blockers reduced territorial aggression in temperate stonechats during the breeding season, but had no influence on territorial behavior in a non-breeding context [8, 71]. A disconnection of territoriality from testosterone outside a breeding context was also reported in red-throated Ant-tanagers *(Habia fuscicauda).* In this neotropical species with high levels of extra-pair matings, territorial take-overs take mainly place outside the breeding season when testosterone levels are low and not during breeding when testosterone levels are high [34].

On a cautionary note, our conclusions on stonechats are based on simulated territorial intrusions with decoys and playback that originated from the overall region of a tested population, but not always from the tested population itself. Studies in other species found a low discrimination ability between local and non-local playback stimuli in males, but a high discrimination ability in females [99, 100]. However, male and female European stonechats have been shown to respond more strongly to playback and decoys from their own than from foreign populations [77]. In our study, behavioral experiments in European stonechats were conducted in Germany, but performed with playback from English populations and decoys from an Austrian population. Thus, we might have underestimated the aggressive response of temperate male stonechats towards simulated territorial intrusions, because using local playback and decoys may have elicited a stronger response. Nevertheless, 82% of all males attacked the decoy, which is higher than the probability of an aggressive response found in a previous study in the same populations where no song playback was used [24]. Furthermore, the latency to attack varied strongly with breeding stage and was lowest during nest-building, which all suggests that the birds responded appropriately. In contrast, in our African populations, we used local playback, but decoys also originated from other African populations. Hence, there was slightly greater local familiarity with the stimuli in African populations, but we may have also underestimated aggressive responses of African stonechats.

Nevertheless, the seasonal variation in attack latency seems to be robust and is very similar in tropical and temperate stonechats. Overall, we find it unlikely that the total lack of differences in aggression between the populations could have arisen from small differences in familiarity.

In contrast to the consistent association between seasonal aggression and seasonal testosterone in stonechats, in our study differences in seasonal changes in song behavior were not associated with those in seasonal changes in testosterone levels. At the beginning of the breeding season European stonechats sang with higher song and element rates and a higher peak frequency than African stonechats, despite similar testosterone levels. Thus, the main function of high levels of testosterone in stonechats and likely in many other tropical and temperate species seems facilitation of high intensity territorial aggression associated with the fertile period of the female. This conclusion is supported by other studies on temperate species that defend territories both in a breeding and a non-breeding context. These studies have shown that in these species song, territorial behavior and testosterone are also at least partially dissociated (e.g. [9, 23]).

Previous generalizations of low testosterone concentrations in tropical birds may have been biased by studies of neotropical or Australian species with extended breeding seasons [28, 101–107]. Long breeding seasons can lead to low breeding synchrony [93] and low competition between pairs [105], potentially making high testosterone concentrations unnecessary. Studies comparing the variation in testosterone concentrations across temperate and tropical latitudes in closely related species or populations are rare. Apart from stonechats, studies have only been published on closely related taxa of the genus *Zonotrichia* that breed in the Neotropics and North America. The life history of neotropical high altitude rufous-collared sparrows *(Z. capensis)* is very similar to the life history of afrotropical stonechats: both are residents and breed seasonally at high altitudes and in open grassland habitats during a relatively short breeding season of 3–4 months [108]. Similar to tropical stonechats, equatorial rufous-collared sparrows have high peak testosterone levels that can be even higher than those of their temperate congeners [38]. However, testosterone implants or androgen blockers had no effect on territorial aggression during breeding [37, 109] – however, the exact breeding stage of individuals was not known. If in tropical seasonally breeding species the main function of testosterone is to support high intensity territorial aggression during female fertility, then testosterone or androgen blocker implants may only be effective during the fertile period of the female.

The differences in song performance which we report for tropical versus temperate stonechats may well be related to differences in territoriality and migratory behavior. Song characteristics play an important role during male-male interactions [56] and for mate attraction [57]. In European stonechats, as well as in many other species, song is under strong sexual selection [77] and may be used to communicate the males’ quality in a reproductive context [75]. At the beginning of the breeding season, populations with seasonal breeding territories, and in particular migratory species, are under temporarily more intense sexual selection than resident populations with year-round territories and may have to invest more into sexually selected traits such as song [61]. Specifically, a high song and element rate and a high peak frequency may accelerate the acquisition of a territory and a mate, and may promote early laying, which is crucial for breeding success in many species [110]. Thus, high performance in these traits at the beginning of the breeding season is presumably particularly important for the reproductive success of European stonechats, which during these stages acquire both, territories and mates. The overall greater song performance of European stonechats fits well with evidence that song performance is increased in response to selective pressures [69]. To the best of our knowledge, only one study so far has compared song structure between tropical and temperate populations within the same taxon: similar to stonechats, neotropical house wrens *(Troglodytes aedon)* sang fewer elements at a slower rate than temperate house wrens [68]. Nevertheless, although selective pressure might not be as high as in temperate migrants, tropical stonechats and several other seasonally breeding tropical species sing more during the breeding season than outside a breeding context [35, 37, 74, 111–114]. To clarify the role of testosterone for song behaviour in stonechats, studies using androgen and estrogen receptor blockers are necessary [115].

In seasonally breeding species, regardless of latitude, short peaks of testosterone may help to avoid costs associated with high levels of this hormone, including potential impairment of the immune system, survival and male parental care [31, 32]. Several mechanisms have been suggested that may allow species to benefit from the positive effects of testosterone on reproductive performance, but avoid the associated costs [31]. As reported here, the peak in testosterone may be restricted to a short period of time (resistance hypothesis, [31]). Additionally, the brain may become insensitive to testosterone outside this brief period of sexual activity (insensitivity hypothesis, [31]). These two mechanisms need not be coupled. For example, in seasonally breeding tropical rufous-collared sparrows, which naturally sustain only a short peak in testosterone, most males stopped taking care of young after they had been implanted with testosterone pellets, indicating that they had not become insensitive to the hormone [116].

In some species with long breeding seasons, testosterone concentrations increase only during social interactions or periods of social instability (social modulation hypothesis, [31]). For example, in tropical spotted antbirds *(Hylophylax n. naevioides)* testosterone concentrations were very low during breeding, but rose transiently during prolonged simulated territorial intrusions [105]. Alternatively, tropical seasonal breeders, like the stonechats in this study, may especially benefit from brief periods of high testosterone levels and low testosterone concentrations throughout the rest of the breeding season. They could reap the benefits, such as prevention of extra-pair copulations, while not threatening the advantage of their slow, tropical pace of life [28], exemplified by greater longevity and extended parental care [81, 117, 118]. Whether tropical male stonechats are also additionally behaviorally insensitive to testosterone during parenting could be tested through testosterone implant studies.

## Conclusions

This is the first study that assessed the role of testosterone, territorial behavior and song concomitantly in several closely related taxa and populations of a songbird breeding at different latitudes from Europe to the Afrotropics. In both European and African male stonechats, a strong territorial response during the fertile period of females coincided with high testosterone concentrations. However, European males sang with a higher song and element rate during the pre-nesting and nest-building phase than African males, despite similar testosterone levels. Thus, in male tropical stonechats seasonal breeding and short breeding seasons may have led to similar peak testosterone concentrations and territorial aggression as in temperate stonechats. However, year-round territories and stable pair-bonds have reduced the pressure of finding a new mate at the beginning of the breeding season in tropical stonechats, which is reflected in a lower song performance in African compared to European male stonechats. Future studies should determine the role of extra-pair paternity in temperate and tropical stonechats as a potential evolutionary driver of high testosterone peaks when females are fertile. Further, as the data collected in this study are correlational, experimental approaches using androgen and estrogen receptor blockers to determine causal relationships between testosterone and behavior are necessary. This kind of information is particularly rare in tropical species, and especially for studies that also investigate song structure. Taken together, our study suggests that breeding in seasonal environments during short breeding seasons is a major selective force for high peak testosterone levels of male birds independent of breeding latitude and variation in pace of life, and that particular behaviors, in our case song performance, can be uncoupled from peak testosterone levels.

## Additional file

Additional file 1: Table S1. Datasets. (XLSX 99 kb)

## Abbreviation

STI: Simulated territorial intrusion
